# Engineered Swine Models of Cancer

**DOI:** 10.3389/fgene.2016.00078

**Published:** 2016-05-09

**Authors:** Adrienne L. Watson, Daniel F. Carlson, David A. Largaespada, Perry B. Hackett, Scott C. Fahrenkrug

**Affiliations:** ^1^RecombineticsSt. Paul, MN, USA; ^2^Masonic Cancer Center, University of MinnesotaMinneapolis, MN, USA; ^3^Genetics, Cell Biology and Development, University of MinnesotaMinneapolis, MN, USA; ^4^Pediatrics, University of MinnesotaMinneapolis, MN, USA; ^5^Center for Genome Engineering, University of MinnesotaMinneapolis, MN, USA

**Keywords:** swine models, preclinical cancer models, genetically engineered swine, cancer genetics, genome engineering

## Abstract

Over the past decade, the technology to engineer genetically modified swine has seen many advancements, and because their physiology is remarkably similar to that of humans, swine models of cancer may be extremely valuable for preclinical safety studies as well as toxicity testing of pharmaceuticals prior to the start of human clinical trials. Hence, the benefits of using swine as a large animal model in cancer research and the potential applications and future opportunities of utilizing pigs in cancer modeling are immense. In this review, we discuss how pigs have been and can be used as a biomedical models for cancer research, with an emphasis on current technologies. We have focused on applications of precision genetics that can provide models that mimic human cancer predisposition syndromes. In particular, we describe the advantages of targeted gene-editing using custom endonucleases, specifically TALENs and CRISPRs, and transposon systems, to make novel pig models of cancer with broad preclinical applications.

## Introduction

**Figure 1:**
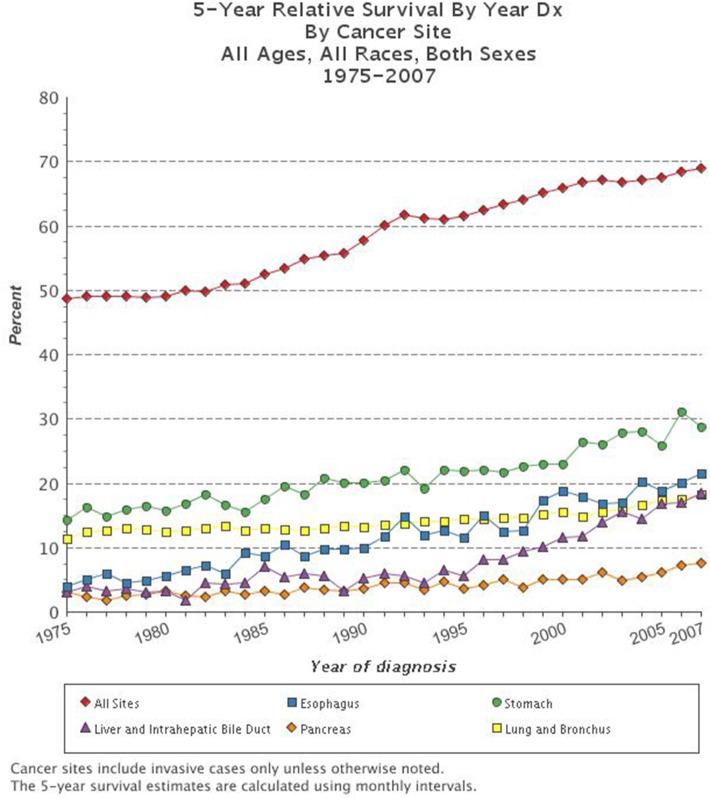
**Five-year survival rate based on cancer type**. The combined 5-year survival rates of all types of cancer have improved over the past decades, yet survival for the most deadly types of cancer including esophageal, stomach, liver and intrahepatic bile duct, pancreatic, and lung and bronchial remain very dismal (www.cancer.gov; N.C.I. Surveillance Research Program).

Cancer is the second leading cause of death in the United States. The National Cancer Institute's Surveillance, Epidemiology, and End Results (SEER) data, estimated that in 2012 there were more than 13.7 million people living with cancer in the U.S. (Siegel et al., 2012). Trends suggest that in 2015 there will be over 1.6 million new cancer diagnoses in the U.S. and a staggering 589,000 deaths due to cancer (SEER). Throughout history there have been dramatic improvements in the methods by which we detect, diagnose, and treat cancer, yet the 5-year survival for all types of cancer remains at a dismal 66.5% (N.C.I. Surveillance Research Program; Mukherjee, 2011; Siegel et al., 2012). While the overall trends in cancer mortality in the U.S. have been reduced over the past decades, there are still several types of cancer for which the prognosis is very poor and for which few improvements have been made (Figure 1). Indeed, it has been suggested that the apparent increase in 5-year survival rates is due to earlier diagnoses, rather than improvements in treatment, for many types of cancer. The lifetime risk of developing cancer in the U.S. is over 40%, which emphasizes the need to better understand this deadly disease and improve outcomes for patients diagnosed with cancer (Siegel et al., 2012).

Cancer is a genetic disease in which cells acquire or inherit mutations, leading to uncontrolled growth of cells in the blood or solid organs (Hanahan and Weinberg, 2011). Animal models, and specifically mouse models, have played a major role in our understanding of the genetic basis of cancer and the role of specific genes and gene mutations in the development and progression of cancer. Mice led the way for the identification of new therapies to treat cancer owing to advances in constructing specific mutants in the late 20th century. Hundreds of mouse models of cancer have been made and studied. However, gaining a complete understanding of cancer, which turns out to be an astonishing number of variant diseases, and translating this knowledge to better treatments and ultimately a cure has been elusive. Clearly, there are limitations to using rodents to model human diseases including large differences in their size, anatomy, physiology, drug metabolism, chromosome structures, and their genetics. Most cancer studies done in mice involve inbred lines of mice in which every locus is homozygous—a condition that inhibits translation of murine studies to humans (Hunter, 2012). To augment studies in the mice, new animal models of cancer are needed. Swine may turn out to be the best alternative models due to their size, physiological, genetic, and biochemical similarities to humans (Prather et al., 2008; Schook et al., 2008; Ganderup et al., 2012; Swindle et al., 2012; Flisikowska et al., 2013; Helke and Swindle, 2013). High-throughput genome sequencing and a collection of precision-genetic tools combined with tools for bioinformatics analysis, and profiling of gene expression/proteomics can be applied in swine. The ability to modify mammalian genomes through transgenesis and targeted nucleases, united with the development of advanced reproductive technologies including cloning, allows researchers to create complex and unique models of cancer in swine that are more applicable to human disease.

## The limitations of rodent models of cancer

Due to the vast differences between rodents and humans, the ability to model the complex diseases such as cancer is quite limited (Cheng et al., 2014). Humans are 3,000 times larger than mice, live 30–50 times longer, and therefore undergo about 10^5^ more cell divisions in a lifetime (Rangarajan and Weinberg, 2003). Without genetic modification, mice develop cancers of mainly mesenchymal origin, such as sarcomas and lymphomas, whereas humans have a bias toward the development of epithelial cancers with age (Rangarajan and Weinberg, 2003). The small size and short lifespan of mice means that loss of certain tumor suppressor genes is insufficient to result in development of cancer in a highly penetrant manner, particularly when such mutations are heterozygous. Accordingly, investigators have used the Cre-Lox system to homozygously inactivate tumor suppressors in a tissue or cell type-specific manner. While this is often sufficient to drive tumor formation, such a situation does not mimic the disease course in patients in which rare loss of heterozygosity (LOH), a genetic condition in which one copy of a gene (or genetic locus, portion of chromosome, etc.) is lost or deleted due to a mutation or chromosomal event, occurs in a field of heterozygous cells. LOH is a common phenomenon in cancer, resulting in homozygous loss of tumor suppressor genes in a subset of cells in the body, often leading to the development of a tumor or the progression of an existing tumor. Because mouse chromosomes are telocentric, LOH often occurs in mouse models by loss of the entire chromosome carrying the wild type tumor suppressor gene allele in cells heterozygous for a tumor suppressor gene mutation (Luongo and Dove, 1996). However, in human tumors LOH usually occurs via sub-chromosomal deletions covering the wild type tumor suppressor gene locus (Thiagalingam et al., 2001; Petursdottir et al., 2004).

On a cellular level, murine cells have a lower threshold for genetic and/or epigenetic changes that lead to transformation in culture, which demonstrates fundamental differences in the mechanistic properties of cancer development between mice and humans (Holliday, 1996). Arguably, the most profound difference between rodent models and humans is the essentially 100% homozygosity of every locus in inbred mouse lines, which may represent only a single individual in the entire population, making extrapolation back to entire human populations challenging (Kaiser, 2015). Mouse cells are immortalized much more readily than are human cells (Rangarajan and Weinberg, 2003). It has also been suggested that mouse cells respond to oncogenic Ras expression in a different manner than human cells; RAS oncogenes require RAL signaling in human cells, whereas the requirement for this signaling pathway is much reduced in RAS oncogene transformation of mouse cells (Hamad et al., 2002). Laboratory mouse strains have very long telomeres, and readily re-express TERT, in contrast to human cells (Holliday, 1996; Kim Sh et al., 2002). Moreover, mice do not develop the same kinds of genetic instability that human cells do during tumorigenesis, perhaps due to their shorter lifespan that could restrict the number of sequential mutations that accumulate in human tumors (Kim Sh et al., 2002).

Many organ systems vary so greatly between rodents and humans that certain types of cancer cannot be accurately modeled. For example, when one copy of the tumor suppressor gene adenomatous polyposis coli (*APC*) is inherited in humans, LOH leads to polyps in the large intestine that progress to invasive carcinoma. In contrast, mice that are heterozygous for *Apc* develop polyps in the small intestine that rarely show disease progression (Karim and Huso, 2013). Such differences in cancer development are due to inherent biological differences between man and rodent and are not limited to the intestinal polyps, but are seen in many mouse models of cancer (Table 1). There are fundamental differences in how tumorigenesis occurs in rodents and humans. This is well illustrated by variations in tumor spectrum when certain tumor suppressor genes, known to cause specific cancers in humans, are knocked out in mice (Table 1). Specifically, the five deadliest cancers in the U.S. (Figure 1) either cannot be modeled in rodents, or have ineffective models for identification of treatments that translate to the clinic.

**Table 1:** **Comparison of knockout mouse models to human patients**.

**Gene**	**KO mouse**	**Patients**	**References**
*APC*	Small intestine polyps which do not typically progress	Large intestine polyps that progress to invasive carcinoma	Groden and Burt, 2012
*BRCA1*	No cancer development	80% risk of breast cancer, 55% risk of ovarian cancer	Evers and Jonkers, 2006
*BRCA2*	No cancer development	80% risk of breast cancer, 25% risk of ovarian cancer	Evers and Jonkers, 2006
*NF1*	Leukemia, pheochromocytoma	Plexiform neurofibromas, malignant peripheral nerve sheath tumors, optic nerve glioma, astrocytoma, leukemia	Gutmann and Giovannini, 2002
*NF2*	Bone tumors, lymphoma, lung adenocarcinoma, hepatocellular carcinoma	Schwannomas, meningiomas, ependymomas	Gutmann and Giovannini, 2002
*RB*	Pituitary tumors	Retinoblasoma, osteosarcomas, prostate, breast cancer	Taneja et al., 2011
*TP53*	Osteosarcoma, soft tissue sarcoma, lymphoma	Breast cancer, brain tumors, osteosarcoma, soft tissue sarcoma	Taneja et al., 2011

The size limitation in rodents makes the development of novel imaging modalities and surgical techniques nearly impossible, yet these are key techniques needed to diagnose and treat a wide variety of tumor types in patients. Moreover, the rate of metabolism is much, much higher in mice compared to humans (Rangarajan and Weinberg, 2003). These differences mean that the pathways by which tumor progression occurs can vary dramatically when comparing mouse models to human cancer. As a consequence, the tumors that develop in a mouse model may respond differently to therapy. For the genetic and physiological reasons, including vast differences in drug metabolism and xenobiotic receptors, rodents also poorly model toxicity, sensitivity, and efficacy when used in preclinical drug studies (Swanson et al., 2004). The ability to establish toxicity and drug sensitivity pre-clinically in animal models is immensely important because less than 8% of cancer drugs translate successfully in Phase I clinical trials from animal models (Mak et al., 2014). While mice have provided numerous insights into the biology of cancer, their historical limitations emphasize the need to develop new models for cancer research, such as swine.

## Advantages of using swine cancer models

The anatomical, physiological, and genetic similarities between swine and humans are striking, suggesting that disease modeling in this large animal may better represent the development and progression of cancer seen in human patients (Swindle et al., 2012). Swine have been widely used in many areas of biomedical research due to such a high resemblance in organ systems. For these reasons pigs are commonly used in cardiovascular research where models of atherosclerosis, thrombosis, and myocardial infarction have been used to understand these health conditions in patients and to develop therapeutic and medical device interventions (Dixon and Spinale, 2009; Vilahur et al., 2011). The similarity in size and anatomy of the swine cardiovascular system allows design and testing of stents and tissue engineering of blood vessels (Bedoya et al., 2006; Gyongyosi et al., 2006). Further, comprehensive studies of the skin, urinary, integumentary, and digestive systems demonstrate extensive similarities to humans (Swindle et al., 2012). This history suggests that swine may be extremely useful as models of human cancer.

Perhaps of greater importance is the degree of genetic variation in pigs, including those used for disease models. Numerous genetically distinct lines of pigs exist and are available for model development, with various levels of diversity and inbreeding. Cultivation and characterization of these lines provides the opportunity to address both basic science and preclinical research needs. Lines that are low in variation provide a predictable platform for the development of therapeutics and toxicology research. But, like patients, many swine herds are highly outbred, with tremendous genetic and phenotypic heterogeneity that is more reflective of the patient population. This heterogeneity has two major consequences. First, genes that act as “drivers” or are otherwise critical to cellular transformation are more likely to be evident in pigs because they must act in the presence of other genetic variations. Also, therapeutic interventions that show efficacy will have to operate in many genetic environments in pigs, likely more accurately predicting safety and efficacy in patients.

The swine genome (*sus scrofa*) has been completely sequenced and, as expected, it shares considerable homology to the human genome (Schook et al., 2005; Groenen et al., 2012). Extensive conservation between pigs and people at the protein and primary sequence level, and extensive chromosomal synteny provide opportunities to address the initiation and progression of cancers, including frequently observed indels, inversions, and translocations, an outcome prohibitive in rodents, where synteny is more fragmented (Schook et al., 2005; Groenen et al., 2012). For instance, one study identified conservation between human and pigs of 112 loci, wherein a human amino acid that is implicated in a human disease is the same in swine (Groenen et al., 2012). Further, gene expression profiling and proteomics have been rapidly advancing in swine (Garbe et al., 2010). With the full genomic sequence in swine, the advancement of bioinformatics tools, the ability to modify somatic swine cells with transgenesis and targeted nucleases, and the development of techniques such as somatic cell nuclear transfer (SCNT), we are now able to create genetically engineered swine models of human disease (Prather et al., 2008). The homology between human and swine genes will be a guide for engineering exact human disease alleles into the swine genome. In the past 4 years, a platform for genetically engineering the swine genome using targeted nucleases and homology-dependent repair (HDR) has been developed (Carlson et al., 2012a; Tan et al., 2013). As described below, this technology allows the replication of precise amino acid changes and/or truncating mutations in oncogenes and tumor suppressor genes known to drive initiation, progression, or metastasis in human cancer. The use of targeted nucleases to engineer swine genocopies, or exact mutant alleles that cause cancer in human patients, represent a more accurate animal model than removing entire exons using standard knockout strategies or overexpressing oncogenes using transgenesis, which have been the mainstays in murine models for decades. Further, recent precision genetic technologies can support the development of single-gene modifications and complex, multiple-gene changes as well as chromosomal translocations in a single generation in swine.

In addition to recent advances in making precise genetic modifications to large animal genomes, there has been significant progress in technologies for testing consequences of genetic changes. Imaging modalities such as computed tomography (CT), magnetic resonance imaging (MRI), and positron emission tomography (PET) can be easily applied to large animals such as pigs, whereas application of analogous clinical protocols is difficult in rodents (Sieren et al., 2014). By applying these imaging modalities to swine models of cancer we can improve detection techniques, better monitor progression, and more accurately measure response to therapy. The size of the pig allows for radiation-directed therapies to be tested and optimized. Surgical resection is the first line of defense and often the standard of care for many cancers. The size of the pig allows for refinement of surgical techniques and studies of local tumor recurrence, both of which are difficult or impossible to perform in rodents. Tumor natural history is an area that is difficult to study in rodents due to their short lifespan, about 1/30^th^ that of humans (Rangarajan and Weinberg, 2003). Swine can live up to 10 years thereby enabling researchers to carefully follow the development of tumors, tumor progression, invasion, and metastasis in the absence of intervention over time. Additionally, the identification of biomarkers may be feasible in these animals due to the facile nature of accessing blood and tissue samples, the abundance of sample material and the ability to perform longitudinal blood sampling over long periods of time. Understanding tumor heterogeneity may be well suited for a large animal, as samples could be collected from many different tumors over time and followed for variations in somatic mutations, gene expression, or differential responses to treatment.

One of the main drawbacks of rodent models of cancer has been their inability to identify safe and effective drugs to treat cancer. Mouse models of cancer have been poor predictors of drug safety, toxicity, and efficacy (Gould et al., 2015). Further, routes of administration in mice are largely limited to intravenous (i.v.), intraperitoneal (i.p.) or oral gavage. Pigs have been widely used in preclinical drug toxicology, and are a standard large animal model for preclinical toxicology prior to human studies (Ganderup et al., 2012). The size and ease in handling pigs allows for drugs to be administered in the same way that patients are given them, including orally, intravenous (i.v.), intraperitoneal (i.p.), inhalation, dermal absorption, subcutaneous, intramuscular, and transmucosal. Longitudinal blood sampling can be performed to assess drug exposure and metabolism over long periods of time, and the amount of blood samples that can be taken from swine, in a short period of time, enhances the ability of pharmacologists to get precise kinetic data following drug exposure. There is significant homology in xenobiotic receptors in swine and human that regulate drug metabolism and pharmacokinetic properties (Myers et al., 2001). The cytochrome P450 (CYP) superfamily of proteins play a critical role in the processing and metabolism of drugs, and again, many studies have shown parallels in the structure and function of these molecules in pigs and humans (Myers et al., 2001). Importantly for pediatric cancer drug studies, juvenile pigs have been shown to have human-similar pharmacokinetic responses to certain drugs that cannot be modeled in other animals (Roth et al., 2013). The use of pigs in preclinical drug testing may identify safer and more effective therapies as well as establish dosing and routes of administration for new drugs prior to human clinical trials. Furthermore, a facile porcine genome engineering platform enables future humanization of drug metabolism in swine models.

## Applications of precision genetics to model cancer in swine

There have been three main types of disease models in swine that have been applied toward cancer modeling—spontaneous, induced, and genetically modified. Spontaneous models of pig cancers are rare, because like humans, pigs develop cancer with age, and as an agricultural animal produced mostly for food, most pigs do not survive to the age where cancer would be commonly seen. Sinclair miniature white swine have been a valuable model of malignant melanoma, which was identified as occurring in these animals in 1967 and has since been selected for by breeding (Oxenhandler et al., 1979). Another study identified 92 cases of leukemia in 3.7 million pigs tested, and 58% of those cases were in pigs under 6 months of age (Anderson and Jarrett, 1968). A range of rarer cancers have been described in older pigs (Brown and Johnson, 1970). However, owing to the economic necessities of keeping costs low in animals of agricultural importance, pigs harboring diseases due to rare mutations are euthanized without further study. Induced models of cancer in swine have been developed and are providing valuable insights into triggers of tumorigenesis found in some agricultural environments. In one study, researchers exposed pigs to N-nitrosodiethylanime to induce hepatocellular carcinoma (HCC) that resembles human HCC (Li et al., 2006). Another induced model took advantage of a naturally occurring severe combined immunodeficiency (SCID)-like Yorkshire pig line and transplanted human melanoma and pancreatic carcinoma cells, demonstrating the usefulness of these animals as human tumor xenografts (Basel et al., 2012).

**Figure 2:**
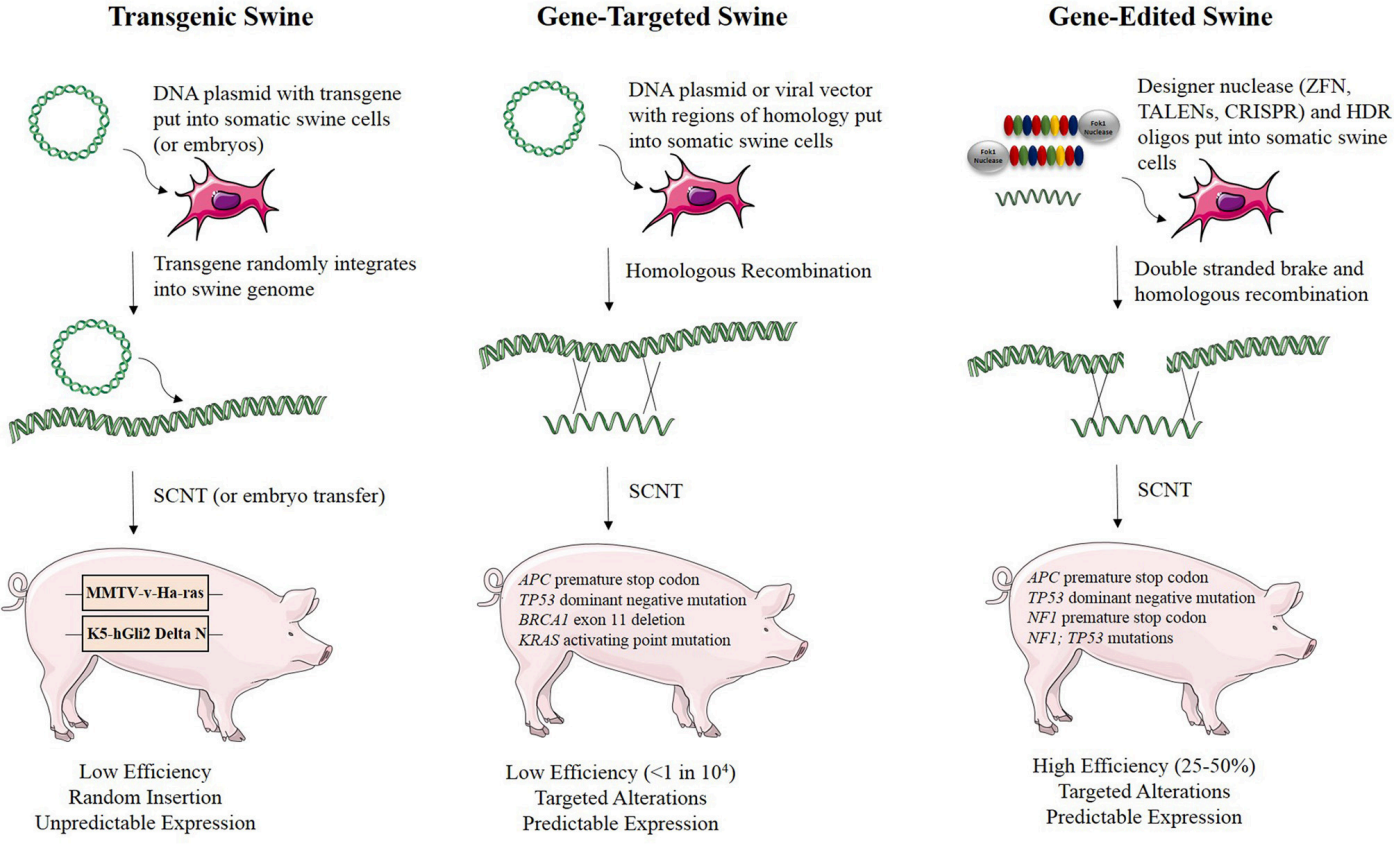
**Methods for developing genetically engineered swine models of cancer**. There are three methods for developing genetically engineered swine. Transgenic swine are created by randomly integrating exogenous DNA into the swine genome, resulting in random insertion and unpredictable expression. Gene-targeted swine are created using large, homologous DNA sequences to integrate exogenous DNA into the swine genome at targeted loci, but this method has low efficiency, often requiring the introduction of some exogenous markers such as drug resistance genes. Gene-edited swine are made using designer nucleases, such as TALENs or CRISPRs, and use a double stranded break at a specific locus to induce efficient homologous recombination to make a targeted alteration in the genome (Yamakawa et al., 1999; McCalla-Martin et al., 2010; Luo et al., 2011; Flisikowska et al., 2012; Leuchs et al., 2012; Sieren et al., 2014; Li et al., 2015).

Genetic engineering and gene editing technologies are being developed for cancer modeling in pigs (Figure 2, Table 2). A transgenic pig carrying the *MMTV-v-Ha-ras* oncogene was developed by microinjection of DNA into embryos, although no tumors developed in these animals (Yamakawa et al., 1999). A basal cell carcinoma model was created by making a transgenic pig in which *Gli2* was expressed under control of a keratinocyte-specific promoter (McCalla-Martin et al., 2010). Standard gene-targeting methods developed in mice have been applied to pigs and led to the development of a familial adenomatous polyposis (FAP) model in which gene-targeting was used to introduce premature termination codons (PTCs) in *APC* by electroporation of linearized vector DNA into mesenchymal stem cells and subsequent SCNT to produce animals (Flisikowska et al., 2012). Similarly, gene-targeting was used to introduce a dominant-negative missense mutation, R167H, in the swine tumor suppressor gene *TP53* using a recombinant adeno-associated virus (rAAV) in fetal fibroblasts, which were cloned to produce animals that when bred to homozygosity for the R167H mutation developed lymphomas, osteogenic tumors, and renal tumors at varying rates (Sieren et al., 2014). These animals represent a great model for humans with germline mutant *TP53* mutations, seen in Li-Fraumeni patients, as they develop hematopoietic malignancies, bone tumors, and adrenal gland tumors in the kidney as well. A breast cancer model was attempted in the pig using rAAV-mediated *BRCA1* knockout, but no phenotype was observed due to death of the animals by unknown causes before they reached 3 weeks of age (Luo et al., 2011).

**Table 2:** **Genetically engineered swine models of cancer**.

**Gene**	**Mechanism**	**Method**	**Phenotype**	**References**
*RAS*	MMTV-v-Ha-ras transgene	Transgenesis	No tumor development	Yamakawa et al., 1999
*GLI2*	K5-hGli2 Delta N transgene	Transgenesis	Basal cell carcinoma-like lesions	McCalla-Martin et al., 2010
*APC*	Premature Stop codon at 1311	Gene-targeting by linearized vector DNA	Low- and high-grade dysplastic adenomas in large intestine	Flisikowska et al., 2012
*APC*	Premature Stop codon at 1061	Gene-targeting by linearized vector DNA	No tumor development at 1 year of age	Flisikowska et al., 2012
*TP53*	R167H dominant negative allele	Gene-targeting by rAAV	Lymphoma and osteogenic tumors	Sieren et al., 2014
*TP53*	R167H dominant negative allele with floxed termination signal	Gene-targeting by vector DNA	TBD	Leuchs et al., 2012
*BRCA1*	Loss of exon 11	Gene-targeting by rAAV	Pigs died by 18 days	Luo et al., 2011
*KRAS*	Floxed G12D activating allele	Gene-targeting by promoter trap gene targeting vector	TBD	Li et al., 2015
*KRAS; TP53*	Floxed, bicistronic *KRAS*^G12D^ cDNA and *TP53*^R167H^ cDNA	Transgenesis	Mesenchymal tumor formation upon AdCre injection	Schook et al., 2015

The ability to activate or inactivate genes in a temporal or spatially-specific manner has been a critical aspect of many murine models of cancer, and this technology is now being developed in the pig by use of the Cre-Lox system (Flisikowska et al., 2013; Schook et al., 2016). There have been three swine models that use the Cre-Lox system for inducible cancer gene expression (Leuchs et al., 2012; Li et al., 2015; Schook et al., 2015). A pig was engineered to harbor a latent *TP53*^R167H^ mutation by the insertion of a transcriptional termination signal between two LoxP sites upstream of the gene, allowing expression of *TP53*^R167H^ only in the presence of Cre recombinase. Cre can be given in a time- or tissue-specific manner to induce recombination and results in the expression of the dominant negative *TP53* allele, and in the absence of Cre Recombinase, the latent form of the gene is a knockout; however, at this time, the effects of this allele *in vivo* have not been reported (Leuchs et al., 2012). A second inducible model was generated using gene-targeting to express a Cre-activated *KRAS* ^G12D^ mutation, although the effect of Cre-induced activation of this allele has yet to be tested in these pigs (Li et al., 2015). Most recently, a transgenic “oncopig” was developed in which a Cre- inducible transgene expressing *KRAS* ^G12D^ and *TP53* ^R167H^ was engineered, in hopes to model the many types of human cancers that have *KRAS* and *TP53* mutations (Schook et al., 2015). Indeed, upon transgene activation, porcine cells were transformed in culture, formed tumors in immunodeficient mice, and led to tumors of mesenchymal origin when activated by AdCre injection directly into these animals (Schook et al., 2015). In addition to using the Cre-Lox system for conditional gene expression, as described in the models above, this system can also be applied to conditional deletions of short coding sequences and used as a strategy for inducing chromosomal rearrangements (Schook et al., 2016).

## Using site-specific nucleases for gene-editing to model cancer in pigs

The use of designer nucleases is the latest technological platform being used to modify the germline of model species. This technology includes zinc-finger nucleases (ZFNs), transcription activator-like effector nucleases (TALENs), and clustered regularly interspaced short palindromic repeats (CRISPRs). All of these methods to engineer precise changes into genomes have been used to make genetically engineered mouse models. In short, site-specific nucleases are designed to bind to user-defined regions of the DNA. ZFNs utilize a zinc finger domain which generally contains 3–6 zinc finger repeats recognizing 9–18 base pairs of DNA (Pabo et al., 2001). TALENs utilize a DNA binding domain contains repeated amino acid sequences, each which harbors a Repeat Variable Diresidue (RVD) (Boch et al., 2009). The RVD sequence gives specific nucleotide recognition, allowing TALENs to bind in a sequence specific manner (Boch et al., 2009). Typically, both ZFNs and TALENs utilize a cleavage domain with a bacterial type IIS restriction endonuclease, FokI, which requires dimerization in order for DNA cleavage to occur, creating even a higher level of specificity for site-specific nucleases (Pabo et al., 2001; Boch et al., 2009). The CRISPR/Cas9 system, derived from the prokaryotic immune system, consists of guide RNA (gRNA) sequences that guide Cas9, an RNA-guided DNA endonuclease, which then cleaves the DNA at these recognition sequences (Jinek et al., 2012; Mali et al., 2013). All three site-specific nuclease systems result in a double stranded break in the DNA, which can be repaired by non-homologous end joining (NHEJ) or homologous recombination (HR) when a repair template is provided. NHEJ often results in small insertions and deletions which can be used to disrupt the function of a gene, where HR allows users to engineer defined genetic changes at specific sites within genome (Carlson et al., 2012a,b; Tan et al., 2012, 2013). These genome engineering systems can be applied to primary cells, which, upon modification, can be used for SCNT to generate animals with germline genetic changes (Carlson et al., 2012a; Tan et al., 2012, 2013). Alternatively, these custom nuclease systems can be applied directly to embryos for *in vivo* modification (Bedell et al., 2012; Tan et al., 2012; Lillico et al., 2013).

The use of custom nucleases to enhance the efficiency of HR in swine has been dramatic from less than 10^4^ using standard HR to rates as high as 25–75% using a recently developed TALEN/homology dependent repair (HDR) platform (Carlson et al., 2012a; Tan et al., 2013). Our group has used CRISPRs and TALENs to engineer several pig models of human disease, including models of infertility and atherosclerosis (Carlson et al., 2012a; Tan et al., 2013). Despite these advantages, a disadvantage to using the various site-specific nucleases, is the potential of undesirable collateral mutations that can accompany those that are desired (Kim et al., 2013; Lin et al., 2014; Mussolino et al., 2014; Frock et al., 2015; Hendel et al., 2015). Because the RNA-guided site-specific platforms (e.g., CRISPR-based) may allow U-G base-pairing, their fidelity may be lower than the protein-based platforms (e.g., TALENs). Consequently, although we have used most of the site-specific nuclease platforms, for fidelity and efficiency, we find TALEN-induced cleavages are the best balance for reliable gene-editing (Carlson et al., 2012a; Tan et al., 2013). TALEN technology allows replication of exact cancer mutations found in patients in pigs. Indeed, we have used TALENs to construct a swine models of the cancer predisposition disease, familial adenomatous polyposis by engineering a premature termination codon in *APC* (Tan et al., 2013).

**Figure 3:**
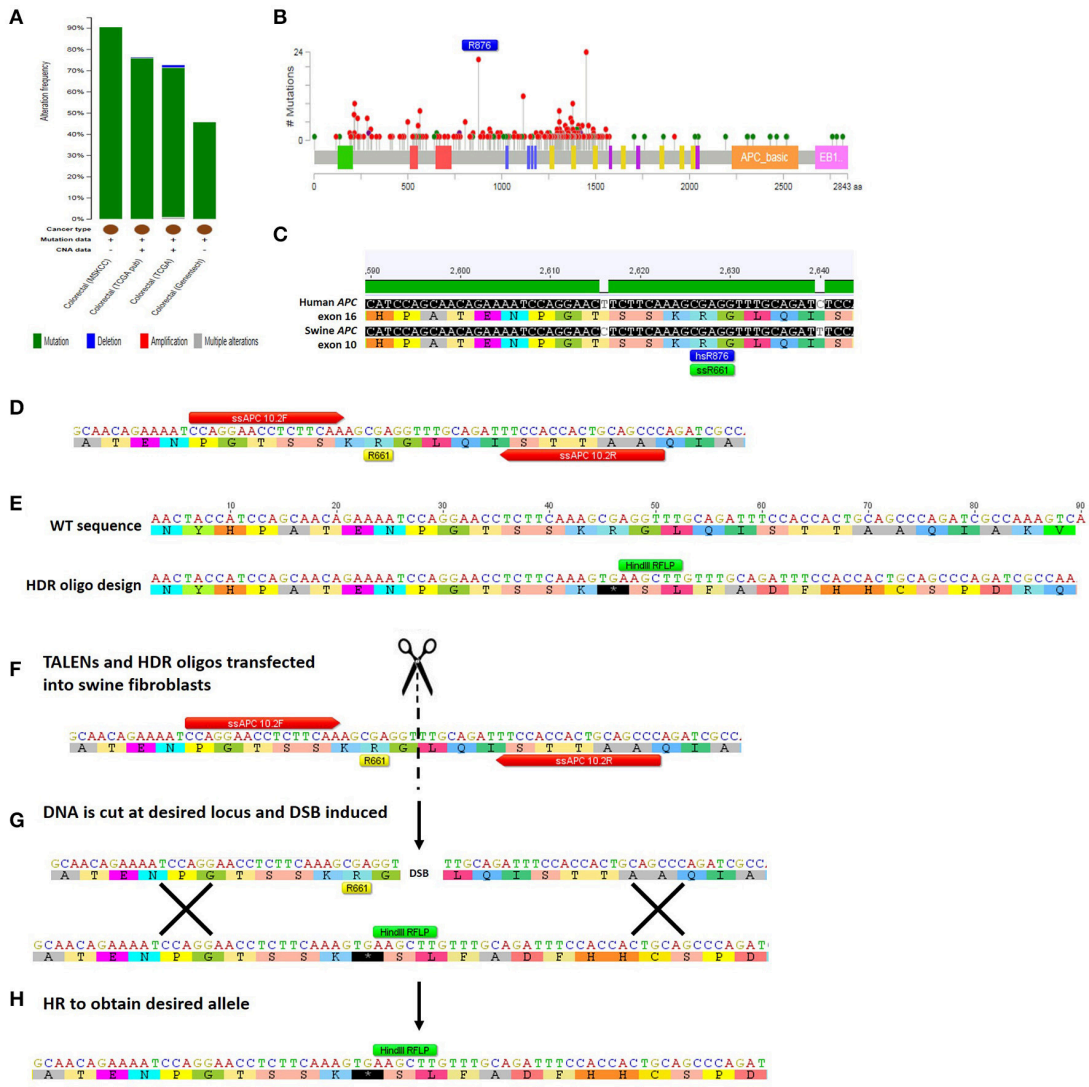
**Method for TALEN-mediated gene-editing to make a swine colorectal cancer model. (A)** The tumor suppressor gene *APC* is commonly mutated in colorectal cancer at a rate of 46–91% depending on which study is assessed (Cerami et al., 2012; Gao et al., 2013). The majority of the mutations are mutations resulting in truncation of APC protein (Cerami et al., 2012; Gao et al., 2013). **(B)** This figure shows the rate of specific mutations across the *APC* gene, with truncating mutations shown in red and missense mutations shown in green (Cerami et al., 2012; Gao et al., 2013). One of the most common mutations in colorectal cancer is an R876X truncation mutation that was seen in 22/727 mutations analyzed (Cerami et al., 2012; Gao et al., 2013). **(C)** Human and swine *APC* genes were aligned and human exon 16 where R876 is found (shown in blue) aligns with swine amino acid 661 in exon 10 of *APC* (shown in green) (Flicek et al., 2014). **(D)** The locus surrounding swine *APC* R661 was inputted into software that identified TALENs that would bind and cleave the DNA near this site (TALEN binding sites shown in red; Cermak et al., 2011; Doyle et al., 2012). **(E)** A 90mer HDR-oligo was designed with homology upstream and downstream of targeted mutation including the TALEN binding sites. A C → T base pair change was introduced to create a premature termination codon. Additionally, four nucleotides were added which would cause a frameshift and a novel RFLP (*Hin*dIII) site (shown in green). **(F)** TALENs and HDR-oligos are then transfected into primary swine fibroblasts. **(G)** TALENs cut at the specified location resulting in a double-stranded break (DSB) and the HDR-oligo acts as a template for homologous recombination (HR). **(H)** HR results in the desired allele with the novel premature STOP colon, RFLP site, and frameshift incorporated into the genome of the swine fibroblasts (Carlson et al., 2012b; Tan et al., 2013).

TALEN-based genome editing for making swine genocopies of human cancer mutations is demonstrated for colorectal cancer in Figure 3. More generally, for a given type of cancer or known cancer driver gene, the method begins with the identification of common mutations within a gene of interest (Figures 3A,B; Cerami et al., 2012; Gao et al., 2013). Second, the location of the human mutation must be identified in the swine gene using bioinformatic approaches in which the amino acid sequence of the human and swine genes are aligned and the mutated human amino acid in humans is identified in swine (Figure 3C; Flicek et al., 2014). Third, custom nucleases such as TALENs are designed to induce a DSB at the appropriate site and HDR-oligonucleotides are designed to introduce the desired mutation (Figures 3D,E; Cermak et al., 2011; Doyle et al., 2012). We can employ a strategy in which the HDR-oligo design includes not only the mutation of interest, but a novel restriction length polymorphism (RFLP) that allows facile screening of a large number of cells for the desired mutation, although introduction of a single point mutation in the absence of an RFLP allele can be engineered as well. Fourth, TALENs and HDR-oligos are transfected into primary swine fibroblasts, where they will cut the DNA and induce HDR to introduce the desired mutation (Figures 3F–H). Transfected cells can be easily screened by RFLP and sequenced to confirm that the intended mutation is present (Carlson et al., 2012a; Tan et al., 2013). In the last step, these cells are used for SCNT to produce gene-edited swine that have the precise human cancer-causing mutation. It should be noted that this technology has also be applied to gene-editing in pig embryos, in addition to somatic cells, avoiding the need for the somatic cell nuclear transfer step (Lillico et al., 2013; Whitworth et al., 2014; Wei et al., 2015).

## Unique opportunities for gene-edited pig cancer models

The ability to engineer specific human mutations into the swine genome is critical for accurately modeling cancer in the pig. While gene-targeting with either rAAV or other vectors has the ability to generate animals with swine genocopies of human disease alleles, these methods generally require the introduction of exogenous DNA into the swine genome in the form of antibiotic resistance genes such as puromycin- or neomycin-resistance since standard gene targeting via HR in swine is very inefficient with rates less than 1 in 10^4^. In contrast, the method of gene editing by employing TALENs and HDR-oligos of ~90 nucleotides is highly efficient, with homologous recombination occurring at an average efficiency of about 45% across several TALEN pairs tested (Tan et al., 2013). This allows facile isolation of cellular clones that are heterozygous or homozygous for the engineered allele of interest.

We identified several types of cancer-causing mutations that can be engineered into swine using TALEN-mediated gene editing (Figure 2). Premature STOP codons can be introduced, resulting in truncated gene products, a common phenomenon for tumor suppressor genes in cancer (Figure 3). Tumor suppressor genes have also been shown to contain point mutations that result in a dominant negative protein product, as is the case for the human *TP53* mutation R175H, which functions as a dominant negative in swine (R167H) and can be introduced efficiently using TALEN-mediated HDR (Leuchs et al., 2012; Sieren et al., 2014). Similarly, oncogene activation can be modeled by introducing point mutations such as *KRAS*^G12V^ using TALEN-mediated HDR. TALEN-mediated gene-editing and HDR repair can be used to create large deletions encompassing one or more genes, which are also seen in several types of cancer, such as micro-deletions of *NF1* seen in Neurofibromatosis Type 1 patients (Pasmant et al., 2010).

In addition to making point mutations using custom nuclease-mediated HDR, TALENs can be utilized to increase the efficiency of HDR with much larger constructs such as HDR templates with homology arms of 750 bp or larger that include LoxP sites flanking large exons. By inducing a double-stranded break, HDR occurs at a much higher efficiency than when plasmids or viral vectors are put into cells alone (Shin et al., 2014). Using TALENs to induce large HDR events has many applications in developing swine models of cancer. Many oncogenes and tumor suppressor genes are somatically mutated and therefore they must be expressed or disrupted in specific tissue types, at specific times. Using custom nuclease-mediated HDR, one could introduce LoxP flanked exons of tumor suppressor genes, or introduce latent oncogenes that would need to be activated by Cre-recombinase to support tissue/temporal regulation. This method allows for the introduction of transgenes at specific loci in the genome thereby avoiding unwanted insertional mutagenesis effects. In order to introduce multiple genetic changes that occur in cancer, TALEN-mediated, site-specific mutagenesis can be used to introduce simultaneous targeted disruption of a tumor suppressor gene along with one or more transgenes for oncogene expression.

**Figure 4:**
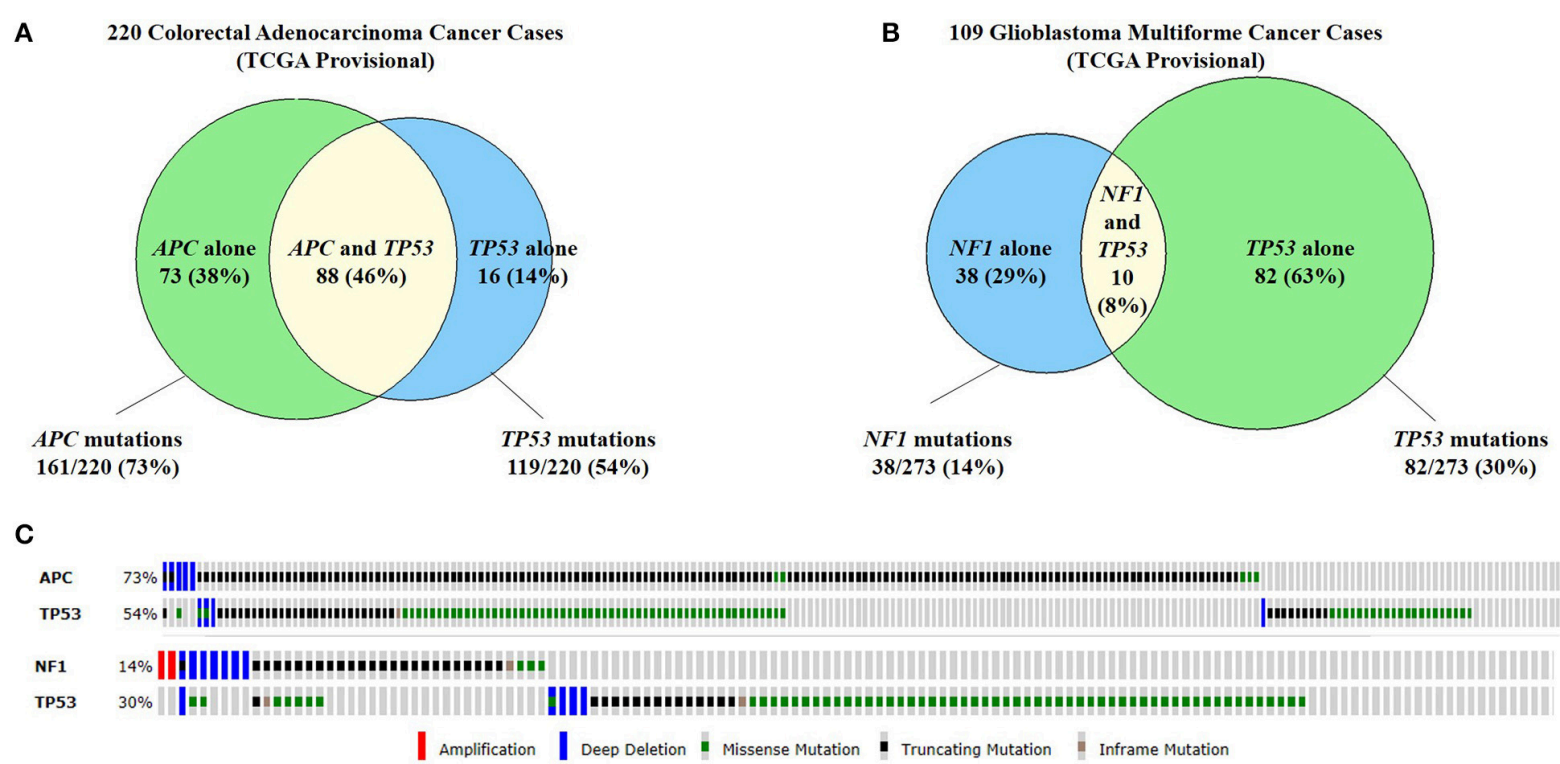
**Commonly co-mutated genes in colorectal adenocarcinoma and glioblastoma multiforme. (A)** A study of 220 colorectal adenocarcinoma cases found that 73% of cases had *APC* mutations and 54% of cases had *TP53* mutations. Forty-six percent of cases had both *APC* and *TP53* mutations. **(B)** Similarly, in 109 cases of glioblastoma multiforme (GBM), 14% of cases has *NF1* mutations and 30% has *TP53* mutations. 8% of cases had both *NF1* and *TP53* mutations. **(C)** The types of mutations seen in *APC* and *TP53* in colorectal cancer (top) and GBM (bottom). Because all of these genes are tumor suppressor genes, the majority of mutations are truncating mutations or missense mutations (Cerami et al., 2012; Gao et al., 2013).

The ability to edit multiple genes at one time is necessary due to multiple genetic alterations in each cancer cell. The efficiency at which the TALEN-mediated gene-editing and small HDR-oligo platform works, allows this technology to be applied toward multiplex gene-editing to model more complex genotypes of human tumors. Epidemiological data and mathematical models in colorectal cancer has suggested that it takes about five to seven rate limiting “steps” for transformation to occur (Renan, 1993). More recently, whole exome sequencing analysis in colorectal and breast cancer has shown that tumors have an average of 90 mutant genes, with 11 of these mutations being “cancer-causing” (Sjoblom et al., 2006). There are many types of cancer for which mutations in two or more genes are clearly demonstrated (Figure 4; Cerami et al., 2012; Gao et al., 2013). Recapitulating this phenomenon would be ideal when engineering animal models of cancer. Developing these multi-hit models is virtually impossible by standard gene-targeting techniques due to such low efficiencies, and would therefore require either serial cloning or animal breeding to obtain multiple alleles in one animal. Both of these alternatives are expensive and time-consuming in large animals such as swine. In contrast, the efficient custom nuclease-stimulated HDR allows the engineering of multiple cancer genes in a single generation.

Multiplex gene editing can also be applied in making models of cancer with associated co-morbidities. Treating patients with cancer becomes quite complex when the patient is suffering from other diseases as well, and this phenomenon of co-morbidity is quite common (Table 3; Sogaard et al., 2013). A study of 15,962 patients showed various types of cancers that are more highly associated with comorbidities (Ogle et al., 2000). The overall frequency of any single comorbidity occurring in this population of cancer patients was 68.7% and 32.6% of patients had two or more comorbidities (Ogle et al., 2000). Some of the effects of these comorbidities on cancer patients and their subsequent survival and cancer treatment are shown in Table 3 (Sogaard et al., 2013). Several studies have demonstrated that patients with comorbidities are less likely to complete chemotherapy, more likely to suffer complications from treatment and/or surgery, and the 5-year mortality hazard ratio for cancer patients with comorbidities ranges from 1.1 up to 5.8 (Sogaard et al., 2013). A multiplex gene editing approach can be taken to model complex disease associations with cancer to understand the impact on survival and treatment approaches. For example, we have used our gene-editing technology to develop swine models of hypercholesterolemia, heart failure, and hypertension and have the ability to generate models of various cancer types in conjunction with these common comorbidities (Carlson et al., 2012a; Tan et al., 2013). Using multiplex gene-editing, it is possible to engineer swine models of cancer in the background of other comorbidity diseases in a single generation.

**Table 3:** **Cancer comorbidities (Ogle et al., 2000)**.

**Comorbidity**	**Common cancers with comorbidity**	**Frequency (%)**	**Consequences**
Cardiovascular Disease (CVD)	Urinary bladder	31.2	• Chemotherapy and radiation can worsen CVD
	Stomach	30.3	• Chemotherapy has cardiotoxic effects
	Lung	30.1	• Concerns with bleeding and thrombocytopenia in patients with stents or prosthetic valves may complicate treatment
	All sites	28.9	
Diabetes	Liver	12.7	• Diabetes significantly increases mortality in cancer patients
	Eye	10.1	• Steroids given with chemotherapy can elevate glucose levels
	Mesothelioma	8.5	
	All sites	6.1	
Hypertension	Colon	46.8	• Hypertension results in an overall higher risk of cancer death
	Eye	44.5	• Hypertension puts patients at risk during surgery and radiation therapy for hypertensive crisis
	Stomach	43.5	
	All sites	41.2	• Chemotherapy and some targeted therapies can increase hypertension
Respiratory Disease	Lung	37.1	• Increased odds of complications with cancer
	Mesothelioma	31.8	• Patients less likely to undergo surgery
	Esophagus	25.1	
	All sites	25.1	
Cerebrovascular disease	Stomach	10.0	• Development of cerebrovascular disease may be provoked by cancer treatment
	Lung	9.1	
	Esophagus	8.5	• Endothelium toxicity and abnormalities of coagulation factors with chemotherapy can induce stroke
	All sites	7.9	
Any single comorbidity	All cancers	68.7	
Any two comorbidities	All cancers	32.6	

**Figure 5:**
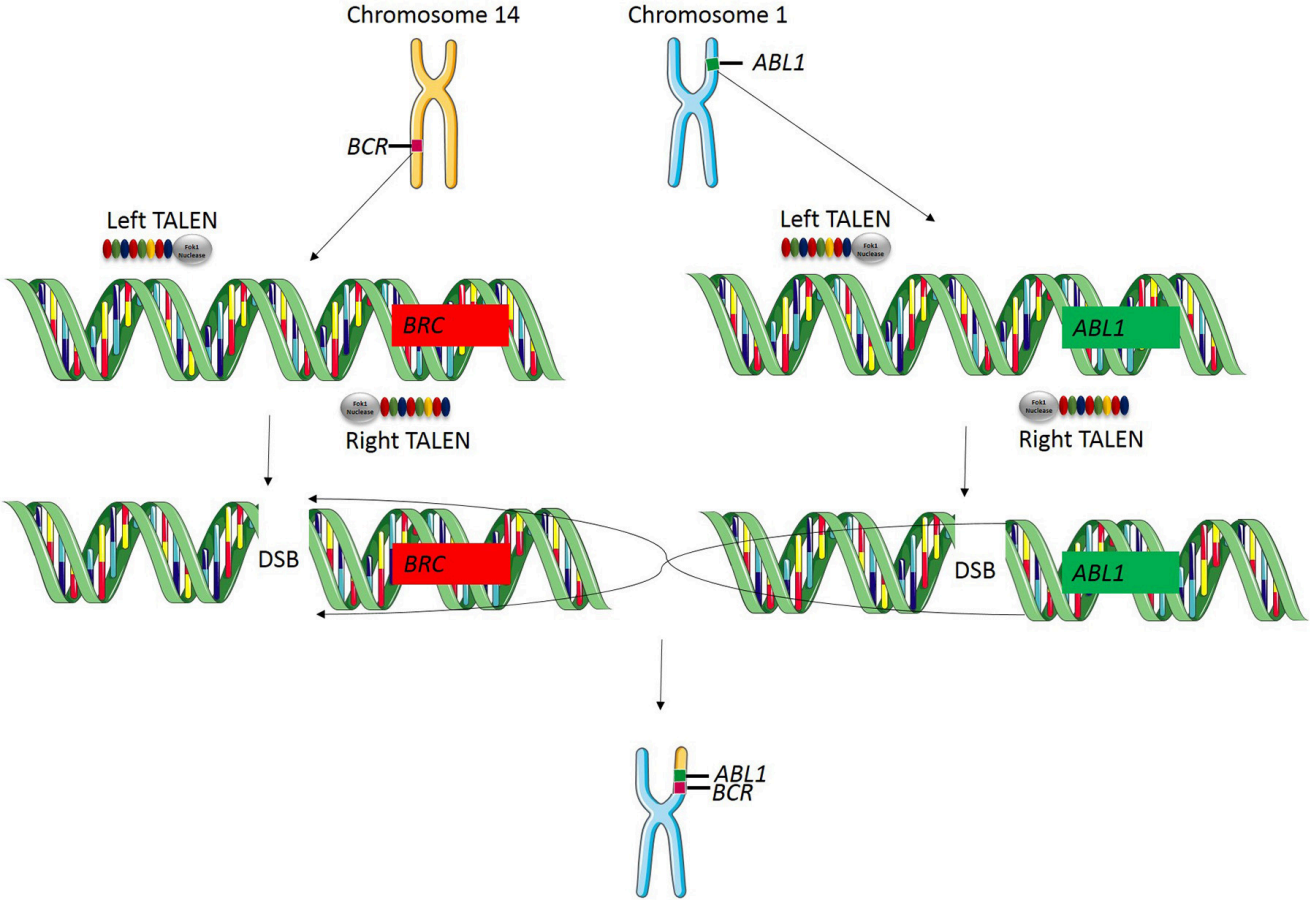
**TALEN-induced translocations**. TALEN technology can be applied for inducing targeted translocations in the swine genome. In this example, a translocation event is known to cause the *BCR-ABL1* fusion gene resulting in chronic myelogenous leukemia. A TALEN pair is designed to cut at the known breakpoint in the *BCR* gene and a second TALEN is designed to cut at the known breakpoint in *ABL1* gene. When these two double stranded breaks occur simultaneously, chromosomal repair will induce a targeted translocation and result in the expression of the *BCR-ABL1* fusion gene at the endogenous loci in the swine genome.

Genomic rearrangements, and specifically, chromosomal translocations are a common occurrence in cancer (Table 4) (Nambiar et al., 2008). The ability to model cancer-causing translocations has been limited to the expression of gene-fusion transgenes in mice and has yet to be demonstrated in swine. Chromosomal translocations can be induced by double-stranded DNA breaks and TALEN technology allows engineering of exact human translocations at endogenous loci in the swine genome resulting in expression from the native promoter (Figure 5). Indeed, cancer translocations have been previously engineered using TALENs and ZFNs in human cells (Piganeau et al., 2013) and could also be applied in porcine cells. Applying TALEN-mediated site-specific mutagenesis to swine opens up a broad field of new research into the mechanism of oncogene activation via genomic rearrangements, the pathogenicity of chromosomal translocations in various cancer types, and investigations into therapies targeting novel gene-fusions and mechanisms of resistance in translocation-driven cancer types.

**Table 4:** **Common cancer-associated chromosomal translocations (Nambiar et al., 2008)**.

**Cancer type**	**Translocation**		**Human location**	**Pig location**
Burkitt's lymphoma	*c-myc*	*IGH@*	*t*_(8;14)(q24;q32)_	*t*_(4;1)_
Follicular thyroid cancer	*PAX8*	*PPARg1*	*t*_(2;3)(q13;p25)_	*t*_(2;1)_
Acute myeloblastic leukemia	*ETO*	*AML1*	*t*_(8;21)(q22;q22)_	*t*_(4;13)_
Chronic myelogenous leukemia/acute lymphoblastic leukemia	*ABL1*	*BCR*	*t*_(9;22)(q34;q11)_	*t*_(1;14)_
Ewing's sarcoma	*FLI1*	*EWS*	*t*_(11;22)(q24;q11.2−12)_	*t*_(9;14)_

## Pigs as models for cancer xenografts

**Figure 6:**
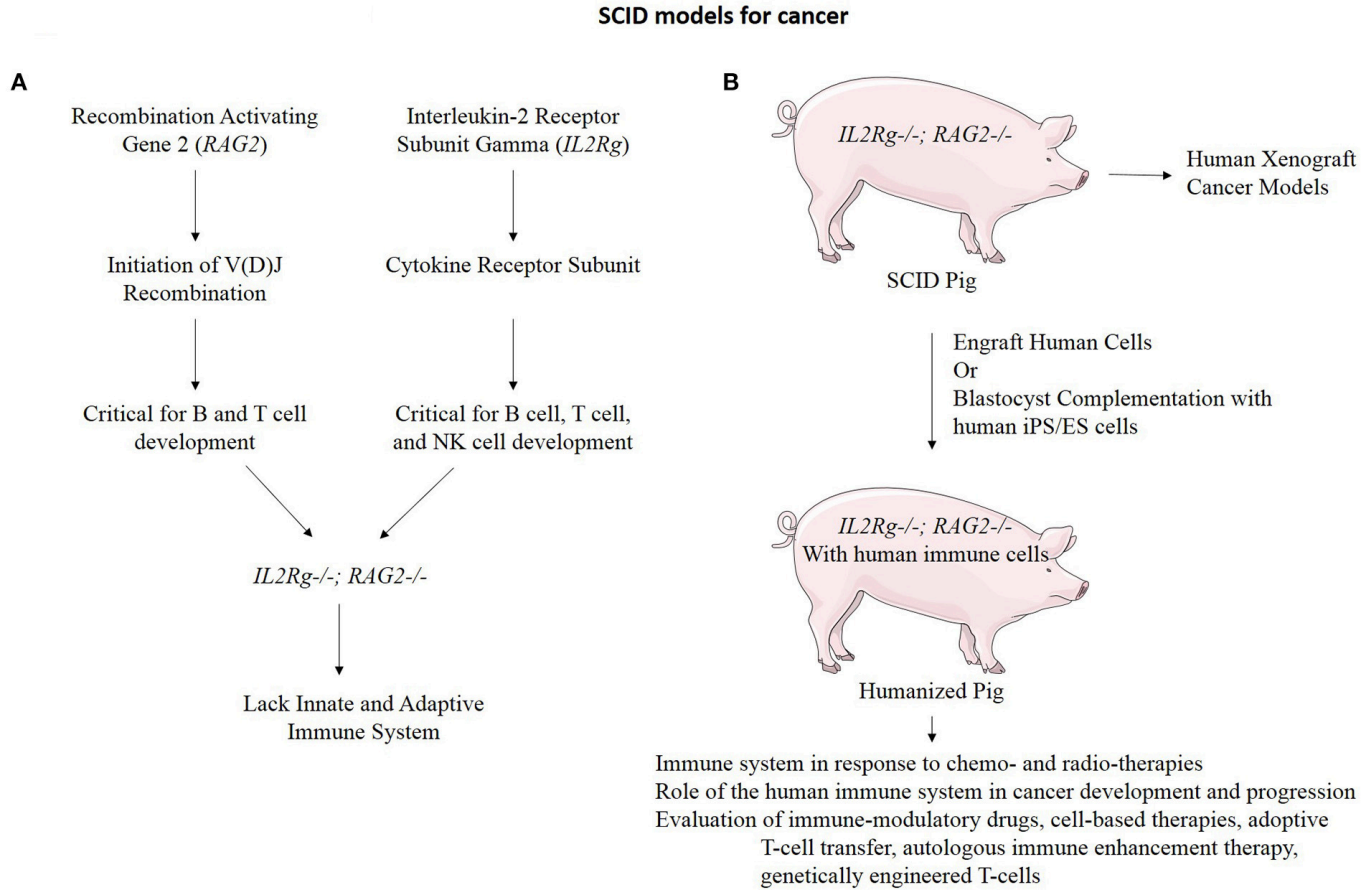
**Immunodeficient swine for cancer research. (A)** *RAG2* and *IL2Rg* function in the development of B cells, T cells, and Natural Killer (NK) cells. When both of these genes are mutated, the resulting organism lacks both the innate and adaptive immune system function (Shultz et al., 2007). **(B)** An *IL2Rg/RAG2* knockout pig to model SCID has many practical applications for cancer research.

An additional application of TALEN-based multiplex gene editing is the ability to simultaneously knock out and add in genes involved in immune system development to facilitate even a broader range of cancer research applications. We and others have developed a severe combined immunodeficiency (SCID) swine model by knocking out genes necessary for both B-cell and T-cell development (Figure 6) (Shultz et al., 2007; Suzuki et al., 2012; Watanabe et al., 2013; Huang et al., 2014; Ito et al., 2014). These animals will allow xenograft experiments to proceed in which one could engraft cells or tissues from human tumors into the pig and monitor these xenografts for growth and development properties, as well as for efficacy studies with novel therapies. These animals can also be engrafted with human immune cells by either blastocyst complementation or transplantation, making a “humanized pig.” This animal would serve several purposes including to investigate the role of the immune system in response to chemo- and radio-therapies for the treatment of cancer and the role of the human immune system in cancer development and progression if combined with a tumor xenograft (Zitvogel et al., 2011). These animals may also have a major impact on immunological research and treatments including the evaluation of: (1) immune-modulatory drugs (Pardoll, 2012; Ileana et al., 2013), (2) cell-based therapies (Fischbach et al., 2013), (3) adoptive T-cell transfer (June, 2007), (4) autologous immune enhancement therapy (Rosenberg, 1984), (5) genetically engineered T-cells (Restifo et al., 2012), and (6) studies of inflammation and infectious disease in the context of cancer (Cibelli et al., 2013).

An alternative approach to using swine as cancer xenograft models is by a method called *in utero* cell transplantation (Fisher et al., 2013). This method relies on the ability of a fetus to become tolerized to foreign antigens by exposing an immunologically immature fetus to xenogenic cells (Fisher et al., 2013). This allows the recipient fetus to recognize human cells as “self,” when the foreign cells are injected prior to population of the pig thymus by CD3+ lymphocytes (Sinkora et al., 2000). This approach has allowed stable and long-term engraftment of both allogenic and xenogenic cells into immunocompetent host animals (Flake et al., 1986; Zanjani et al., 1992a,b, 1994). Using this method, one could perform *in utero* injections of a xenograft cell line of interest, tolerizing the host pig, and allowing for engraftment of human cancer cells in the pig post-natally. The xenogenic cells could be established human cancer cell lines, or human cells engineered with specific mutations and/or transgenes to determine their contribution to tumor formation, tumor growth, or drug responsiveness.

## Applying transposon systems in pigs to study cancer development, progression, immune response, and drug resistance

Transposable elements have been widely used in in forward genetic screens to identify genes involved in cancer, as well as in reverse genetic studies to produce transgenic animals and determine the contribution a gene or set of genes makes in the development of cancer (Tschida et al., 2014). Transgenesis via transposon systems have produced transgenic mice, rats, fish, frogs, and more recently, pigs (Clark et al., 2007; Carlson et al., 2011; Garrels et al., 2011; Jakobsen et al., 2011). Using cytoplasmic or pronuclear injection, transposon systems can efficiently deliver genes of interest into the porcine genome, allowing for the development of transposon-mediated transgenic porcine cancer models (Carlson et al., 2011; Garrels et al., 2011).

Another application of transposon systems in swine is to utilize the ability of transposon mutagenesis systems to screen for genes involved in cancer. Historically, these studies have been done in mice in which one chromosome contains a concatemer of transposons, and upon expression of transposase, these transposons randomly integrate throughout the genome (Moriarity and Largaespada, 2015). By random chance, certain cells will have the right combination of oncogenes activated and/or tumor suppressor genes inactivated to cause a tumor to form (Moriarity and Largaespada, 2015). These studies depend on the development of many, many tumors, which are then sequenced to determine genes that were activated or inactivated by transposon mutagenesis (Moriarity and Largaespada, 2015). Bioinformatics analysis can predict which genes are potential drivers in the development of cancer, because they will undergo mutagenesis at a rate higher than would be expected by random chance (Moriarity and Largaespada, 2015).

The pig offers unique opportunities for applying transposon mutagenesis screens. For example, due to the large size of the pig, one could engineer a swine model harboring transposon concatemers and expressing a transposase, and look for tumors by imaging using MRI, CT, PET, or ultrasound analysis. By using sophisticated imaging techniques, coupled with the large size of the pig and availability of tissue samples, one could biopsy tumors, and look at transposon insertion sites over time to determine tumor evolution genes involved in development, progression, and metastasis (DeNicola et al., 2015). Further, pigs that develop tumors could be treated with drugs, and their tumors monitored over time to look for tumor regression followed by development of resistance. Tumors that show initial regression to a certain drug could be sequenced to identify genes involved in drug sensitivity. Similarly, tumors that develop resistance to a drug or therapy could be sequenced to identify genes involved in the development of drug resistance. Lastly, new and innovative applications of transposon mutagenesis screens can be applied to swine models of cancer. For example, as described previously, the Sinclair miniature white pig develops a spontaneous form of malignant melanoma (Oxenhandler et al., 1979). Interestingly, these pigs show a nearly 100% spontaneous regression of cutaneous melanomas (Oxenhandler et al., 1982). This spontaneous development and regression of melanoma model is a perfect opportunity to utilize a transposon mutagenesis system to identify genes involved in the suppression of regression. Lastly, methods for transposon-mediated transgene delivery to somatic cells have been developed in the mouse and could be applied in the pig as well (Wiesner et al., 2009).

## Pig models of cancer: unanswered questions and looking forward

Cancer remains the second leading cause of death in the U.S. There is a chronic need to understand the etiology and biology of this collection of diseases as well as identify new treatments. The anatomical, physiological, and genetic variations between mice and humans limit the prospects of meeting the needs of patients by modeling cancer in rodents. However, for any novel animal model to be useful in cancer research, it must be adopted and fully tested in many laboratories under many circumstances. Even though pigs may turn out to be better models to investigate cancer and potential therapeutics, the considerable expense associated with large animals over their extended lifetimes coupled with the perceived need to run experiments under various experimental and controlled conditions may impede the rate of their widespread adoption into mainstream science. That will be determined by the scientific community plus funding and private entities that support cancer research and therapeutic development. As a large animal with striking similarities in anatomical structure, physiological function, and genetic makeup to humans, we expect the pig will become an improved model animal to advance decades of cancer research studies conducted in rodents. Custom endonucleases, such as TALENs, coupled with cloning, enable the engineering of swine genocopies of human cancers mutations, providing a myriad of unique and exciting opportunities in cancer research that may ultimately better model cancer seen in human patients and lead to novel biological insights into the mechanism of cancer and more effective treatments for patients.

## Author contributions

AW provided intellectual content and drafted the review article, figures and tables. DC, DL, PH, and SF contributed significant ideas and intellectual content including conception and design and provided critical review and editing.

### Conflict of interest statement

All authors are owners and employees of Recombinetics Inc. Additionally, DL is a consultant, co-founder, and equity holder of NeoClone Biotechnology, Inc., a company providing antibodies to it's customers. DL is a consultant, co-founder, and equity holder of Discovery Genomics, Inc., a company pursuing human gene therapy. DL is a consultant, co-founder, and equity holder of B-MoGen Biotechnologies, Inc., a gene delivery and gene editing company. DL has a collaborative research agreements with Novartis and Genentech. Genentech is funding a research project DL's laboratory.
